# Supplementary material for: The Bio-Safety Concerns of Three Domestic Temporary Hair Dye Molecules: Fuchsin Basic, Victoria Blue B and Basic Red 2
Source: Molecules. 2019 May 5;24(9):1744. doi: 10.3390/molecules24091744 (PMC6539679; doi:10.3390/molecules24091744)
Supplement: Supplementary file 1 [file molecules-24-01744-s001.pdf]

Supplemental Materials

The Bio-Safety Concerns of Temporary Hair Dye Molecules: Percutaneous Absorption, Cytotoxicity, BSA and DNA Interaction Research

Bing Liu[1,2], Shu-fang Jin[3], Hua-Chao Li[1,2], Xiang-Yu Sun[3], Si-Qi Yan[3], Shu-Jun Deng[3] and Ping Zhao[3,*,]

[1] Guangzhou key laboratory of construction and application of new drug screening model systems, Guangdong Pharmaceutical University, Guangzhou 510006, China; liubing520@gdpu.edu.cn (B. Liu)

[2] Key Laboratory of New Drug Discovery and Evaluation of ordinary universities of Guangdong province, Guangdong Pharmaceutical University, Guangzhou 510006, China

3. School of Chemistry and Chemical Engineering, Guangdong Pharmaceutical University, No. 280, Waihuandong Road, Education Mega Centre, Guangzhou 510006, PR China

* Correspondence: pingzhao@gdpu.edu.cn (P. Zhao).

Captions

Fig. S1: The emission spectra of BSA under different concentrations of (a) BR2 and (b) VBB. The arrows indicate the spectra change with the addition of concentration.

Fig. S2: The H-bonds between BSA and dyes, (a) FB, (b) BR2 and (c) VBB based on the lowest free binding energy.

Fig. S3: The binding details between CT-DNA and dyes, (a) FB, (b) BR2 and (c) VBB based on the lowest free binding energy.

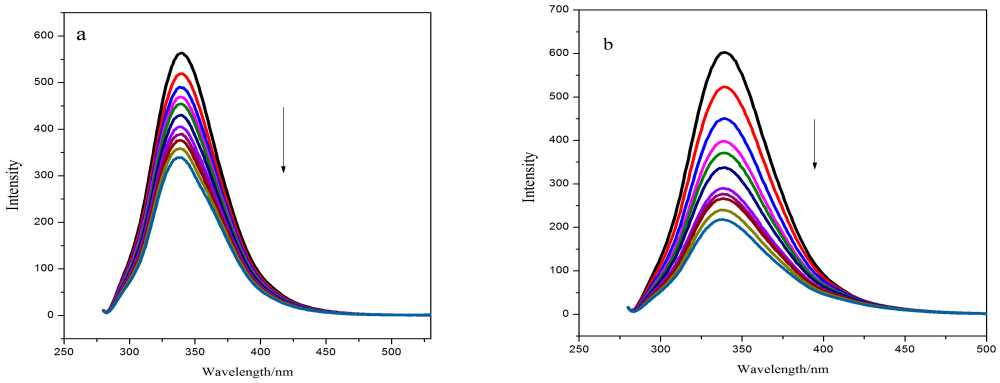

Fig. S1: The emission spectra of BSA under different concentrations of BR2 (a) and VBB (b). The arrows indicate the spectra change with the addition of concentration.

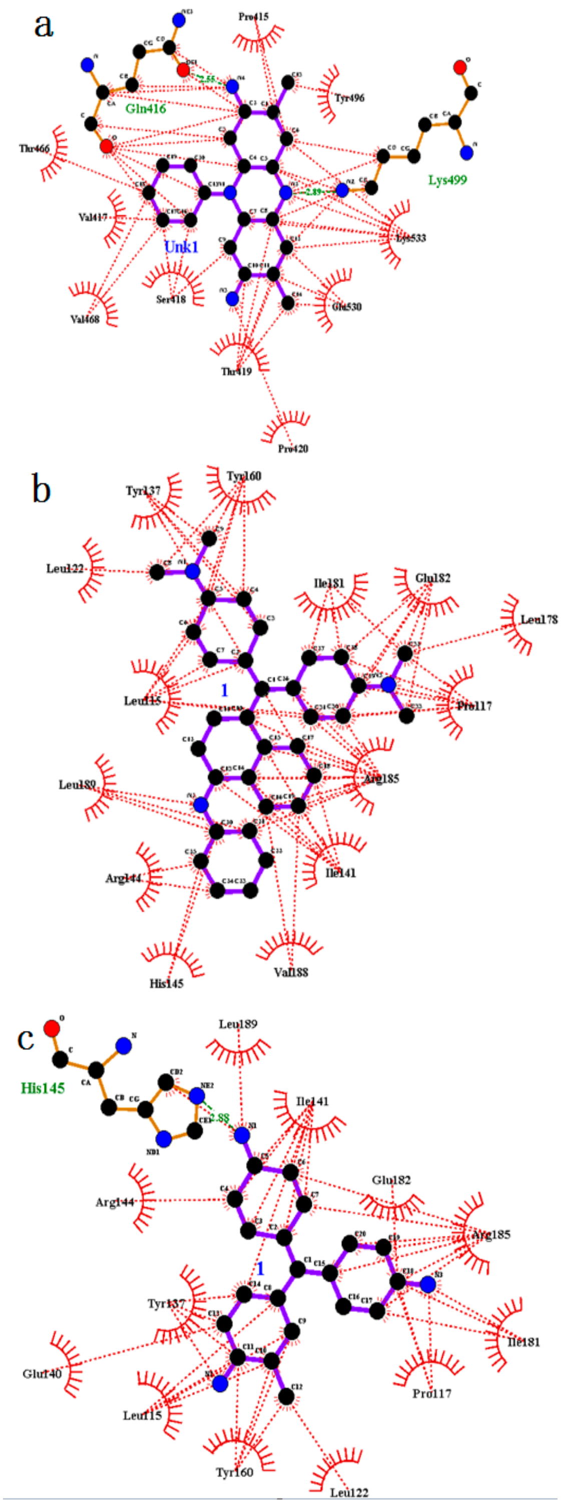

Fig. S2: The H-bonds between BSA and dyes, (a) FB, (b) BR2 and (c) VBB based on the lowest free binding energy.

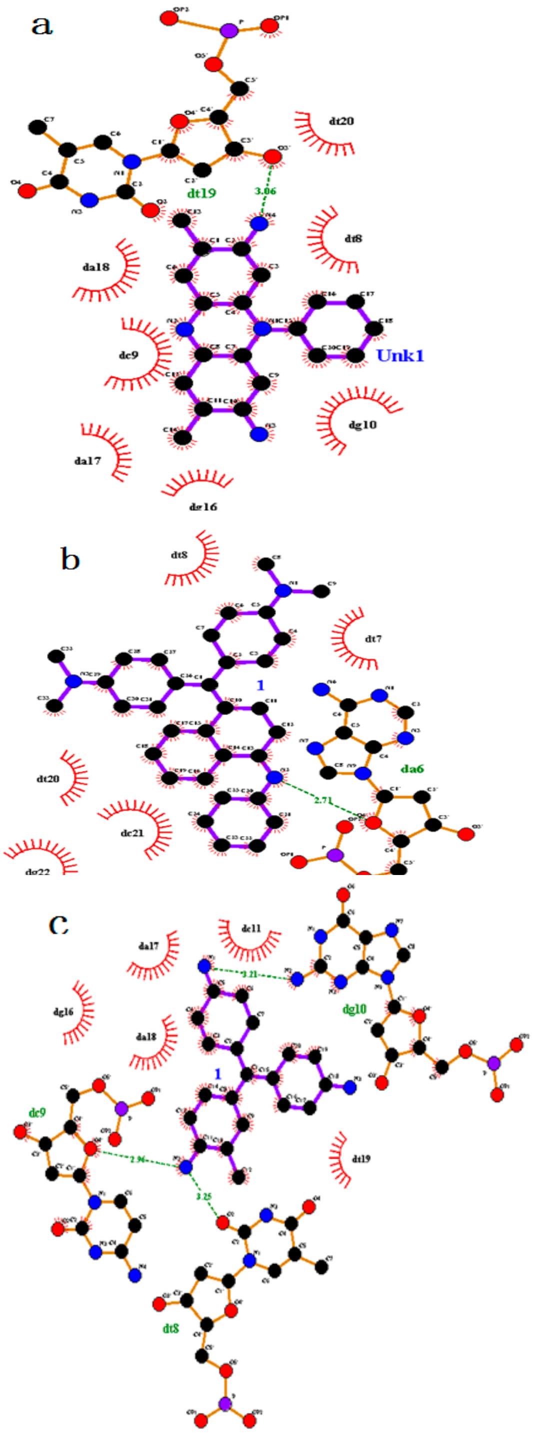

Fig. S3. The binding details between CT-DNA and dyes, (a) FB, (b) BR2 and (c) VBB based on the lowest free binding energy.
